# How many words are Australian children hearing in the first year of life?

**DOI:** 10.1186/s12887-020-1946-0

**Published:** 2020-02-03

**Authors:** Mary E. Brushe, John W. Lynch, Sheena Reilly, Edward Melhuish, Sally A. Brinkman

**Affiliations:** 1 Telethon Kids Institute, University of Western Australian, Level 15, 31 Flinders St, Adelaide, South Australia 5000 Australia; 2 School of Public Health, University of Adelaide, Level 9, Adelaide Health & Medical Science Building, 57 North Terrace, Adelaide, South Australia 5005 Australia; 3 Population Health Sciences, Bristol Medical School, University of Bristol, First Floor, 5 Tyndall Avenue, Bristol, BS8 1UD UK; 4 Menzies Health Institute Queensland G40 Griffith Health Centre, Griffith University, Level 8.86 Gold Coast Campus, Mount Gravatt, Queensland 4222 Australia; 5 Department of Education, University of Oxford, 15 Norham Gardens, Oxford, OX2 6PY UK

**Keywords:** Parent talk, Inequality, Early childhood development, Language

## Abstract

**Background:**

There is evidence that parents from more socioeconomically disadvantaged backgrounds engage in fewer verbal interactions with their child than more advantaged parents. This leads to the so-called, ‘30 million-word gap’. This study aims to investigate the number of words children hear and the number of vocalizations children produce in their first year of life and examines whether these aspects of the early language home environment differ by maternal education.

**Methods:**

Mothers were recruited into a five-year prospective cohort study and categorized into either high or low maternal education groups. Data was derived from the first two waves of the study, when the children were six and twelve months old. At both waves, children were involved in day-long audio recordings using the Language Environment Analysis software that provided automatic counts of adult words spoken to the child, child vocalizations and conversational turns. Descriptive results are presented by maternal education groups.

**Results:**

There was large variation within each maternal education group, with the number of adult words spoken to the child ranging from 2958 to 39,583 at six months and 4389 to 45,849 at twelve months. There were no meaningful differences between adult words, child vocalizations or conversational turns across maternal education groups at either wave of data collection.

**Conclusions:**

These results show that a word gap related to maternal education is not apparent up to twelve months of age. The large variability among both maternal education groups suggests that universal interventions that encourage all parents to talk more to their child may be more appropriate than interventions targeted towards disadvantaged families during the first year of life.

## Background

The early years are fundamental in ensuring children grow up to be healthy, functioning adults [1–3]. By the time children start school there is a clear social gradient in most areas of child health and development [4]. The first five years of life, especially for children from disadvantaged backgrounds, are crucial in overcoming the intergenerational transmission of inequality, such that disadvantaged parents have disadvantaged children, who themselves go on to be disadvantaged adults [5].

Language ability is a critical developmental milestone that is directly related to later literacy, educational attainment and labor market experience. In the Australian context, results from the 2018 Australian Early Development Census, a triennial census of children’s development at age 5, showed that 6.6% of children were developmentally vulnerable on the language and cognitive skills domain and 8.2% were vulnerable on the communication skills and general knowledge domain [6]. Both domains were socioeconomically patterned, with the highest levels of vulnerability amongst children from the most disadvantaged backgrounds. Poorer language skills have been shown to strongly predict poorer education outcomes in the mid and long term [3, 7].

Given socioeconomic inequalities in language development can be detected early and predict later outcomes, mechanisms for enhancing children’s development require further investigation. Currently, some evidence suggests that the amount of maternal language heard during the early years may mediate the association between social disadvantage and child language ability [8].

Numerous studies indicate that parents from more socioeconomically disadvantaged backgrounds engage in fewer verbal interactions with their children, compared to those from advantaged backgrounds [9–13]. The most influential study of language spoken to the child in the home was that of Hart and Risley (1995) involving 42 families from Kansas, USA. From the age of 12 to 36 months, children of parents on welfare, working-class and professional backgrounds heard 620, 1250 and 2150 words per hour, respectively. Within group trends were linearly extrapolated to estimate that by the age of four, children from professional backgrounds heard over three times more than children from welfare families. Thus, the idea of the ‘30 million word gap’ came into being.

Despite the enormous attention the study has received (google hits = 58,800,000), there are clear limitations. First, the study uses a small convenience sample (*n* = 42) and includes only six families on welfare. Second, the data collection method (researchers videotaping one hour per month in the home) is not likely to be representative of the natural home environment. For instance, while unbeknown to the authors at the time, it was later discovered that early evening, when videotaping usually occurred, is a period of extremely high talk for families [11]. Finally, the study began collecting data when children were 12 months of age, neglecting critical language experience under twelve months.

Since the Hart and Risley study, new speech recognition technology called Language ENvironment Analysis (LENA) has become available to allow researchers to objectively measure the amount of parent talk children hear in the home, without the need for videotaping or manual transcription. Gilkerson and colleagues [11] utilized LENA to replicate the work of Hart and Risley with 329 English-speaking families in Denver, USA with children aged 2 to 48 months. Their socioeconomic groups were based on mother’s highest level of completed education, with education groups defined by completed some high school education, completed high school or general education diploma, completed some college and completed bachelor’s degree or higher. Their cross-sectional findings estimated a 4 million word gap by age four between the highest and lowest socioeconomic group, significantly smaller than Hart and Risley’s findings.

Another recent study involved 42 children aged 18 to 48 months from five communities across America with different levels of socioeconomic backgrounds (poor, working-class, middle-class) and like Hart and Risley, captured the number of words heard in the home through videotaping and transcription [14]. The authors main finding showed no meaningful differences between the poor, working-class and middle-class communities in the number of words spoken by the primary caregiver to the child, with some poor and working-class communities showing an advantage in words spoken, compared with middle-class communities. They posit that community variation in the amount of speech addressed to the child cannot be predicted by socioeconomic status alone [15]. This paper questioned the validity of the original Hart and Risley findings, provoking discussion around the importance of the original 30 million word gap hypothesis [15, 16].

The Language in Little Ones (LiLO) study is a prospective cohort study which aims to advance knowledge in this area by combining the use of the LENA software, recruiting a large socio-economically diverse sample, and beginning when children are six months old. The present study aims to quantify the number of adult words that are spoken to the child, number of child vocalizations, and number of times the adult and child engage in a conversational turn over a day, when children are aged six and twelve months. Furthermore, the study aims to examine whether these aspects of the early language home environment differ by maternal education.

## Methods

### Study design

The LiLO study follows two cohorts of children; *a baby cohort* that involves families with a child aged six months old at first data collection and *a toddler cohort* involving families with a child aged three years old at the beginning of data collection. Both cohorts are followed once every six months until the children turn 4 years old. The design includes purposive stratification by two levels of maternal education (only completed secondary school education or less and completed a bachelor’s degree or higher) to explicitly maximize and adequately power contrasts across maternal education groups. At each six-month milestone, families undertake day-long (16-h) audio recordings and complete standardized questionnaires. Families were compensated with a $10 supermarket voucher after each wave of data collection. This paper reports on data from the first and second waves for the *baby cohort.*

### Participants

Recruitment occurred between April 1, 2017 and January 31, 2019 both pre- and postnatally across Adelaide and Port Pirie in South Australia, Bunbury in Western Australia and Gold Coast, Queensland. Pregnant women were approached at Adelaide public hospitals while waiting for their antenatal appointments. Postnatally, mothers were asked to participate at Child and Family Health Service sites during drop-in clinics and at early parenting groups across Adelaide, Port Pirie and Bunbury. Mothers were also approached at council-run immunization clinics, children centres, playgroups and shopping centres across all locations. Recruitment was limited to families whose home language was English. Mothers with a bachelor’s degree or above were recruited into the high education group, and mothers with school only education were recruited into the lower education group. The study also excluded children with diagnosed causes of language impairment (e.g., hearing impairment, Down Syndrome, Cerebral Palsy) and was confined to singleton children and those born full term (37+ weeks) between January 1, 2017 and December 31, 2017.

**Fig. 1:**
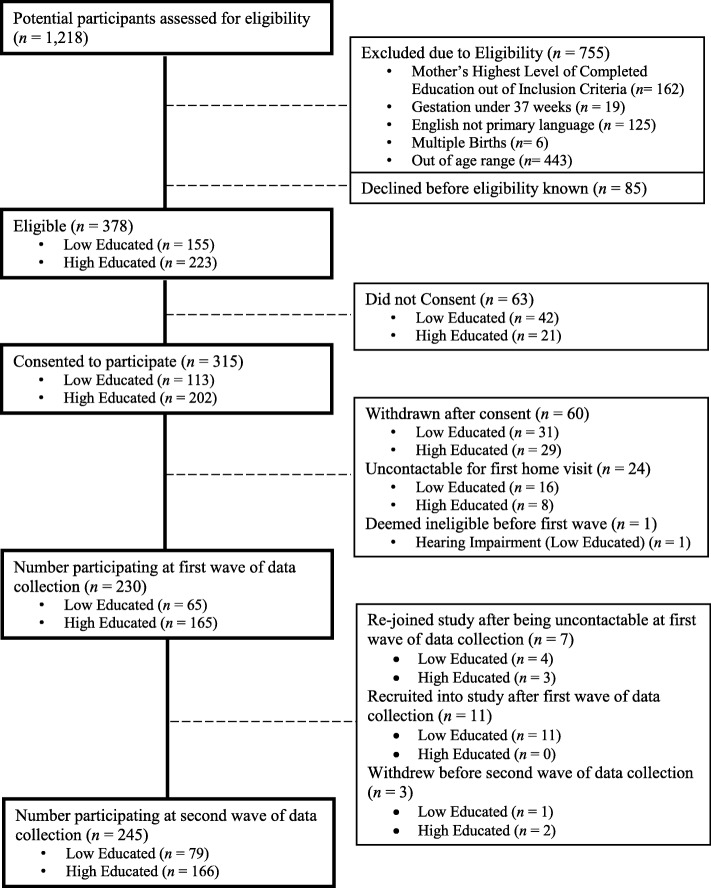
Flow chart of recruitment numbers

A total of 230 families were involved in the first wave and 245 families in the second wave of data collection which included 60.84% of eligible mothers approached (See Fig. 1 for a flow chart of recruitment numbers). Our original power calculations required 120 children in each of the maternal education groups at wave one in order to detect a 0.3SD effect size. Due to the challenges in finding and engaging sufficient mothers with lower education levels we did not meet these initial sample size requirements and therefore extended original recruitment timelines and locations to boost numbers, which meant mothers were still able to join the longitudinal study even if they had missed the first wave of data collection.

### Measures

Families’ natural home language environments were captured using the Language Environment Analysis (LENA) system [11, 17, 18]. The LENA system comprises a specially designed age appropriate vest or t-shirt with a pocket in the front to hold a digital language processor (DLP) with LENA software, which automatically processes the audio captured in the DLP through algorithmic analysis of the speech signal [19]. LENA produces estimates of three key measures used in the current study: adult word counts (AWC), child vocalization counts (CVC) and conversational turn counts (CT). AWC’s estimate the number of adult words spoken in approximately a 10-ft radius of the child wearing the recorder [11]. AWC’s do not necessarily have to be child-directed speech but are loud enough to register on the LENA DLP. CVC’s comprise the number of any speech-related sound made by the child wearing the DLP with each vocalization separated by 300 ms of silence. Finally, CT’s are the number of alternations within a conversation between adult and child vocalizations as occurring within at least 5 s of each other. Either child or adult may initiate the conversation. Reliability testing conducted by the LENA Foundation found a high degree of agreement between human-transcribers and LENA system classification based on 70 h of recording data. For classification of adult words the two raters agreed 82% of the time and for child vocalizations they agreed 76% of the time [18]. It should be noted when overlapping speech occurs in the audio, the LENA software does not categorize this into either adult or child speech. While a trained human-transcriber may be able to identify the primary speaker, the LENA Foundation argues it is not known whether an infant or toddler would be able to distinguish during noisy language input. Therefore it is argued that the exclusion of these segments of audio by the LENA software may provide a more accurate representation of the child’s meaningful language environment [18].

Additionally, during the home visit, the primary caregiver answered questions about family demographics, government payments received by the family, child care arrangements, services accessed by the child and family, and activities in the home with the focus child.

### Procedure

#### Data collection

During data collection a researcher attended the family’s home where they provided the LENA equipment, showed parents how to use it and then asked the standardized questionnaires. The family was given two weeks to complete one LENA recording day. The families were asked to pick a day (to undertake the recording) when the focus child was not in child care or sick, and not when the family had a big event (e.g., sporting match or birthday party). A researcher then returned to the family home after the recording day, picked up the LENA equipment and provided the family with their reimbursement. This procedure was consistent across all families and waves of data collection.

### Statistical approach

**Fig. 2:**
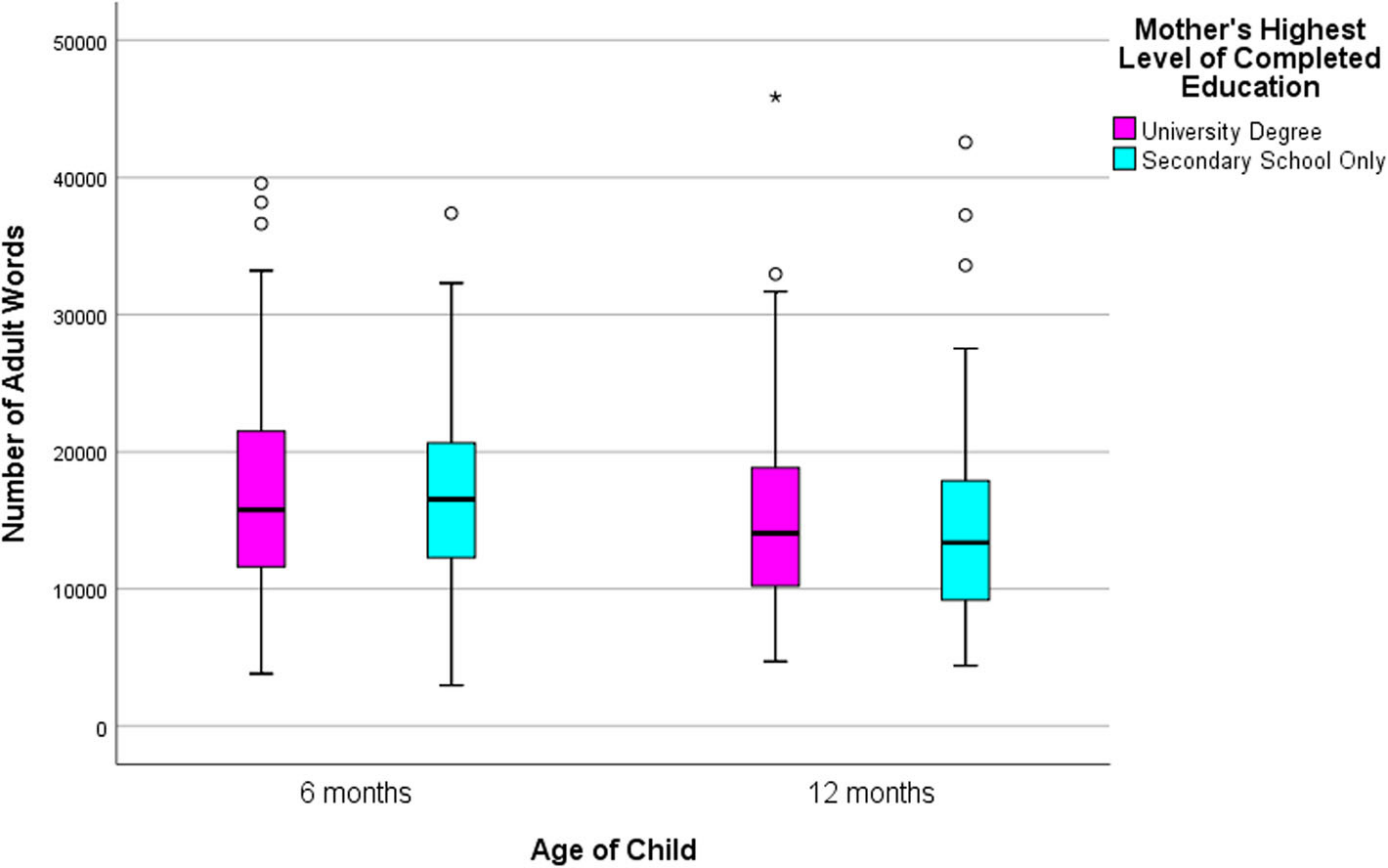
Adult word count at 6 and 12 month wave of data collection by maternal education

**Fig. 3:**
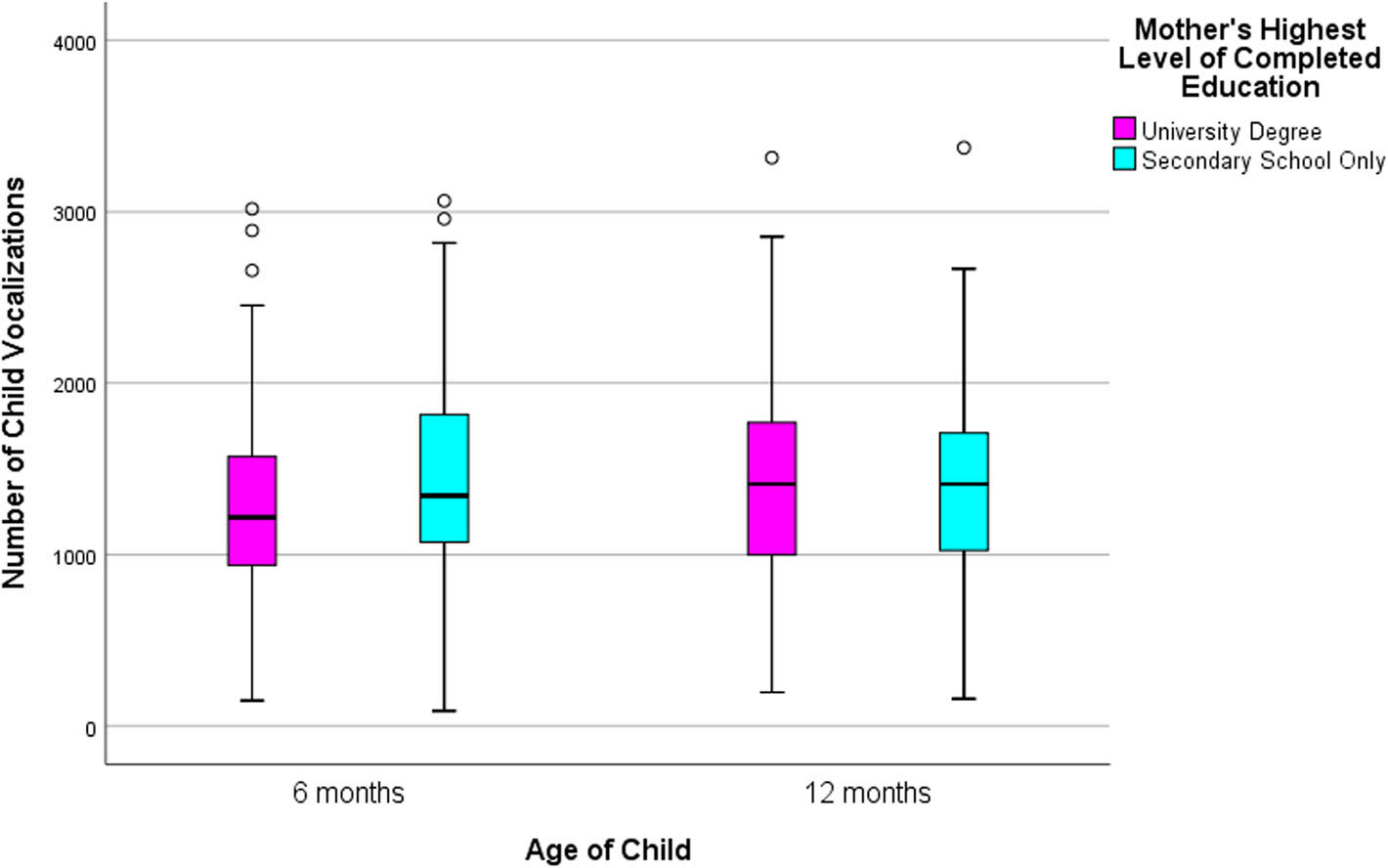
Child vocalization count at 6 and 12 month wave of data collection by maternal education

**Fig. 4:**
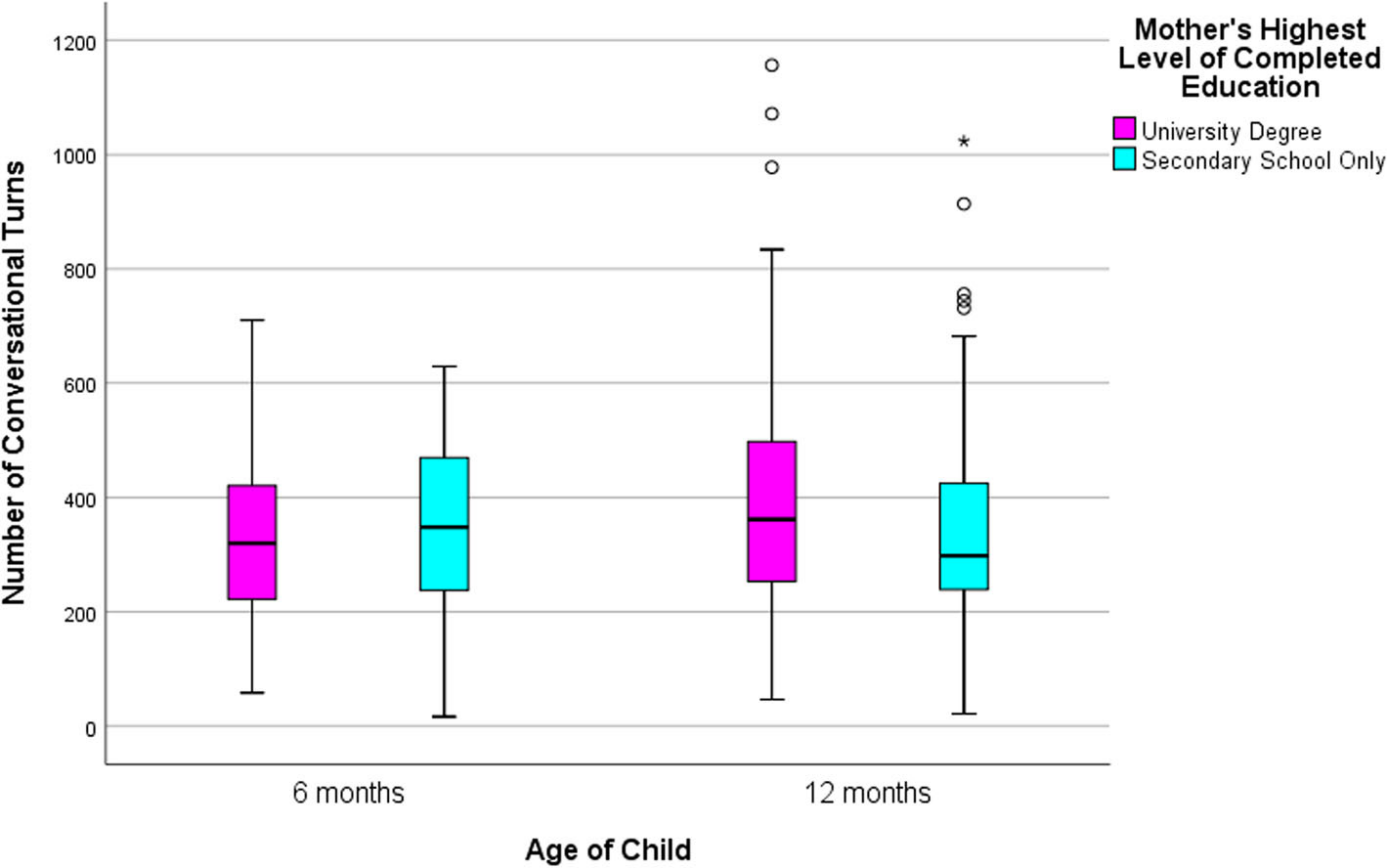
Conversational turn count at 6 and 12 month wave of data collection by maternal education

Descriptive statistics are presented in Table 2 and box and whisker plots in Figs. 2, 3 and 4 to compare the distributions in talk by low and high education groups. The line in the middle of the box represents the median, the bottom of the box represents the 25th percentile and the top of the box represents the 75th percentile. The whiskers of the plot extend to 1.5 times the interquartile range, with outliers falling outside this denoted by an asterisk, and fall at least 3 times outside the interquartile range. Independent sample t-tests were also conducted to compare the means between high and low educated groups. All analyses and graphs were conducted using IMB SPSS version 25.0 [20].

## Results

LENA recordings for the first wave were completed between the August 1, 2017 and July 31, 2018 and recordings for the second wave were completed between February 1, 2018 and January 31, 2019. Each participant family undertook a LENA recording day within two months after turning 6 months and 12 months. Parents rarely used their ability to pause or stop the recording early, with 98.23% of families completing a full 16-h recording day during the first wave and 97.55% of families during the second wave. Of the ten families that stopped the recording across both waves, six completed at least 10 h of recording and noted the recording was stopped as the child went to sleep, therefore was included in the total sample. Three families in the first wave and one family in the second wave completed less than 10 h of recording due to either device malfunction or choosing to stop the recording early and were excluded from further analysis.

The final analysis sample involved 227 families, with 164 in the high education group and 63 in the low education group for the first wave, and 245 families, with 166 in the high education group and 79 in the low education group for the second wave (See Table 1). Note recruitment continued between wave 1 and 2, consequently the larger sample in wave 2. For the first wave, children were aged between 5 and 8 months of age (mean = 5.81) and 53.3% were female. Mother’s average age at birth was 31.34, with 87.7% working until their pregnancy and 56.4% of children being first-born infants. In the second wave children were aged between 11 and 14 months (mean = 11.99) with the same percentage of females.

**Table 1:** Sociodemographic Characteristics of the Sample

	6 month Data Collection (*N* = 227)	12 month Data Collection (*N* = 245)
Child		
Age, mo, mean (SD)	5.81 (0.57)	11.99 (0.51)
Girls, n (%)	121 (53.3)	130 (53.06)
Gestation, wk., mean (SD)	39.2 (1.36)	39.14 (1.34)
Firstborn, *n* (%)	128 (56.4)	131 (53.47)
Mother		
Highest level of completed education, University, *n* (%)	164 (72.2)	166 (67.76)
Age at childbirth, y, mean (SD)	31.34 (4.42)	31.24 (4.57)
Working up until pregnancy, yes, *n* (%)	199 (87.7)	211 (86.12)

As shown in Table 2 there were small differences between the average number of adult words spoken, child vocalizations and conversational turns for the low and high education groups, at both waves. By standard criteria for ‘statistical significance’ children in the low education group vocalized more (approximately 160 vocalizations) than those in the high education group at the first wave (6 months). However, this difference was greatly reduced at the second wave (12 months).

**Table 2:** Daily LENA measures: distribution by the total sample and maternal education

	Adult Word Count				*p*-value*	Child Vocalization Count				*p*-value*	Conversational Turn Count				*p*-value*
	M	SD	Min	Max		M	SD	Min	Max		M	SD	Min	Max	
6 month Data Collection															
Total Sample (*n* = 227)	16,845.88	7102.57	2958	39,583		1319.48	551.74	87	3064		330.77	137.23	16	710	
Mother’s Education															
Low Educated (*n* = 63)	16,747.75	7228.62	2958	37,397	0.90	1454.06	660.86	87	3064	0.05	347.90	152.39	16	629	0.28
High Educated (*n* = 164)	16,883.58	7075.57	3795	39,583		1267.78	496.24	147	3017		324.19	130.84	58	710	
12 month Data Collection															
Total Sample (*n* = 245)	14,888.66	6782.11	4389	45,849		1416.37	561.13	158	3373		369.26	178.34	21	1156	
Mother’s Education															
Low Educated (*n* = 79)	14,407.25	7156.63	4389	42,545	0.44	1406.57	588.38	158	3373	0.85	345.71	188.99	21	1023	0.15
High Educated (*n* = 166)	15,117.76	6606.38	4701	45,849		1421.04	549.45	196	3316		380.46	172.51	46	1156	

*p-value is based on independent sample t-tests comparing the means between high and low educated groups

The plots in Figs. 2, 3 and 4 depict the spread of the data demonstrating enormous variation within the two education groups across both waves. As an example, at the first wave the minimum AWC for the low educated group was 2958 words per day and the maximum count was 37,397 words (mean = 16,747.75; SD = 7228.62). The minimum AWC for the high educated group was 3795 words and the maximum were 39,583 words per day (mean = 16,883.58; SD = 7075.57). This highlights there is little difference between education groups but high variability within education groups and this is consistent for all three LENA measures, revealing high and low adult and child talkers within both education groups.

## Discussion

The purpose of this study was to characterize, for the first time, the amount of talk/ vocalizing Australian children are hearing and uttering at home in the first 12 months of life. The study also examined differences linked to maternal education in adult words, child vocalizations and conversational turns. First, results showed high variability in the whole sample on all three measures of talk when children were six and twelve months of age. However, this did not substantively differ by maternal education. While there may be other factors in the home environment that are associated with this variability such as cultural or emotional characteristics, socioeconomic characteristics indexed in this case by maternal education did not differentiate the three measure of talk. Second, adults in the home of the low education group were talking, on average, just as much as adults in the high education group. In fact, within both education groups, the variability demonstrates some families speak over 35,000 words to their child in a day and others speak less than 4000 words. The similarities between the education groups are also reflected in the number of conversational turns between adults and children over the day, with no meaningful differences between education groups and again high variability in both groups.

The study by Gilkerson and colleagues, is most comparable to the current study and reports a 4 million word gap by age four [11]. Their observations began when children were two months old and they have reported their mean AWC’s, CVC’s and CT’s at 6 months of age (*n* = 50). When they conducted their study, the LENA system only recorded 12-h days, compared to our 16-h recordings. Comparing average word counts for Gilkerson et al. and the LiLO study showed adult words were 1041 vs 1052, for child vocalizations 82.28 vs 82.46 and conversational turns 20.16 vs 20.62 respectively. While these average counts per hour are almost identical in the two studies, Gilkerson and colleagues did not report counts by socioeconomic groups at 6 or 12 months of age, so we are unable to compare [11]. The differing definitions of maternal education groups and different educational contexts in Australia and the United States may partially account for why the current study did not find the difference between education groups that other researchers have reported.

The Language in Little Ones (LiLO) study is the first study with a large sample using objective measures to characterize the verbal home environment by maternal education groups in the first year of life. These findings have important implications for interventions that aim to reduce the word gap, suggesting services with this specific aim may need to utilize a universal approach, rather than simply targeting families from low socioeconomic backgrounds, as it is clear from our data there are adults across both socioeconomic groups who would be considered low talkers. While our data cannot yet explain if the amount families talk to their children in the home will lead to differences in future development outcomes, previous research has suggested this is the case [8, 21, 22]. As the LiLO study progresses, it will describe the trajectories of AWC, CVC and CT’s for low and high maternal education groups and consequences for child development outcomes over the first five years of life.

A shortcoming of the current work is the uneven sample across the education groups, with fewer low educated mothers participating than originally planned. This results from less mothers identified as eligible for the low educated group at recruitment sites and also the lower participation rate into the study for this group. As the LiLO study is longitudinal, attempts to overcome this flaw in future waves will continue by recruiting low educated mothers into our study as it progresses.

A further limitation is that the LENA data cannot effectively capture the quality of verbal interactions, beyond the use of conversational turns. While understanding the context of the words spoken to the child is not the focus of the study, the importance of the quality of early language input for child outcomes is recognized. Nonetheless, if the study can demonstrate the link between parents’ talk and impacts on children’s future development, this can inform the increasing number of interventions using the LENA technology to provide feedback to parents on their quantity of words [23, 24].

## Conclusion

The results from the first two waves of the Language in Little One’s study found large variability within maternal education groups and no meaningful differences between maternal education groups for the number of words spoken by adults to the child or the number of conversational turns between adult and child in the first year of life. This finding has implications for the 30 million word gap hypothesis, suggesting either a word gap does not emerge until after twelve months of age or for children living in Australia the gap does not exist. Implications of these findings suggest that interventions aiming to encourage parents to talk more to their child in the first year of life should be accessible for all parents, regardless of education level.

## Data Availability

The datasets generated and analysed during the current study are not publicly available due to lack of informed consent for data sharing at the time of collection, but are available from the corresponding author on reasonable request. For further information on the data and materials used in this study, please contact the corresponding author.

## Abbreviations

AWC: Adult Word Count
CT: Conversational Turn
CVC: Child Vocalization Count
DLP: Digital Language Processer
LENA: Language ENvironment Analysis
LiLO: Language in Little Ones;
